# Combating Ebola with Repurposed Therapeutics Using the CANDO Platform

**DOI:** 10.3390/molecules21121537

**Published:** 2016-11-25

**Authors:** Gaurav Chopra, Sashank Kaushik, Peter L. Elkin, Ram Samudrala

**Affiliations:** 1 Department of Chemistry; Purdue Institute for Drug Discovery; Purdue Institute for Inflammation, Immunology, and Infectious Disease; Purdue Institute for Integrative Neuroscience; Purdue Center for Cancer Research, Purdue University, West Lafayette, IN 47907, USA; 2 Department of Biomedical Informatics, Jacobs School of Medicine and Biomedical Sciences, University at Buffalo, Buffalo, NY 14203, USA; sashankk@Buffalo.edu (S.K.); elkinp@buffalo.edu (P.L.E.); 3 Department of Internal Medicine, Jacobs School of Medicine and Biomedical Sciences, University at Buffalo, Buffalo, NY 14203, USA

**Keywords:** drug repurposing and discovery, multitarget docking, compound–proteome interaction, candock

## Abstract

Ebola virus disease (EVD) is extremely virulent with an estimated mortality rate of up to 90%. However, the state-of-the-art treatment for EVD is limited to quarantine and supportive care. The 2014 Ebola epidemic in West Africa, the largest in history, is believed to have caused more than 11,000 fatalities. The countries worst affected are also among the poorest in the world. Given the complexities, time, and resources required for a novel drug development, finding efficient drug discovery pathways is going to be crucial in the fight against future outbreaks. We have developed a Computational Analysis of Novel Drug Opportunities (CANDO) platform based on the hypothesis that drugs function by interacting with multiple protein targets to create a molecular interaction signature that can be exploited for rapid therapeutic repurposing and discovery. We used the CANDO platform to identify and rank FDA-approved drug candidates that bind and inhibit all proteins encoded by the genomes of five different Ebola virus strains. Top ranking drug candidates for EVD treatment generated by CANDO were compared to in vitro screening studies against Ebola virus-like particles (VLPs) by Kouznetsova et al. and genetically engineered Ebola virus and cell viability studies by Johansen et al. to identify drug overlaps between the in virtuale and in vitro studies as putative treatments for future EVD outbreaks. Our results indicate that integrating computational docking predictions on a proteomic scale with results from in vitro screening studies may be used to select and prioritize compounds for further in vivo and clinical testing. This approach will significantly reduce the lead time, risk, cost, and resources required to determine efficacious therapies against future EVD outbreaks.

## 1. Introduction

The 2014 Ebola epidemic was caused by a divergent strain of the Zaire Ebola Virus [1] and is believed to have affected more than 28,000 individuals globally, with an estimated mortality of 74% in confirmed Ebola cases [2]. The mainstay of EBV prevention and treatment is infection control precautions and supportive care to the affected individual(s) in order to maintain cardiovascular function while their immune system mobilizes an adaptive response. Considering the complexity [3] and cost [4] of developing a new drug combined with the fact that countries worst affected were also among the poorest in the world, finding an alternate cheaper route for future EVD outbreak treatments is of paramount importance.

Traditional approaches to drug discovery are highly specific to single targets (molecules and indications), focusing on a limited set of interactions between individual protein targets and small molecule compounds, but applying the resulting treatments universally to all patients. The goal generally is to target an essential protein responsible for pathogenesis so as to completely inhibit its function, and then determine its toxicity or side effect profile for human use. Almost all current drugs have been developed by this approach. However, the number of novel drugs being discovered every year has been reduced to a handful. Currently, less than 50 new drugs are approved each year, and most of them are analogues to other existing drugs or other patent workarounds [5]. The estimated average costs for developing a novel drug and bringing it to market can be up to $2.6 billion [6]. Thus, there is a dearth of novel drug development, which is time- and cost-prohibitive [7,8,9,10], particularly for rapidly emerging indications such as divergent strain EVD outbreaks or neglected indications such as orphan diseases [11].

**Figure 1:**
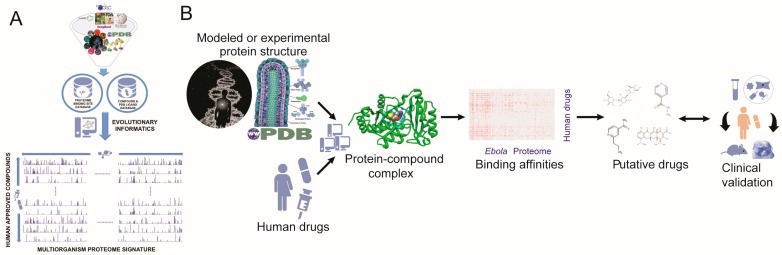
The Computational Analysis of Novel Drug Opportunities (CANDO) platform as applied to five Ebola proteomes. (**A**) General version of the platform used to determine drug behavior and similarity by performing a virtual screen to predict interactions between “all” known drugs and “all” protein structures; (**B**) CANDO platform as applied to Ebola, where the known drugs are docked to structures of five Ebola proteomes to identify the strongest multitarget inhibitors. Credit: Vignettes derived from Protein Data Bank (PDB) structures depicting Ebola virus glycoprotein, matrix protein, nucleoprotein, and nucleocapsid proteins (PDB identifiers 3csy, 4ldd, 4qb0 and 2i8b, 3vne, 3fke respectively).

One solution is to repurpose and reposition existing drugs that are relatively benign in terms of side effects for new indications [11,12,13,14,15,16,17,18,19]. We were one of the first to propose shotgun drug repurposing for malaria based on computational multitarget docking with dynamics [15]. Since then, we have validated our predictive models numerous times [9,16,17,18,20,21,22]. This repurposing can be made more accurate by considering variations (mutations) in proteins encoded by individual genomes. Systematic exploration of drug repurposing opportunities is hindered by extensive competition in the pharmaceutical industry. We utilize this repurposing paradigm along with a computational platform we have developed that evaluates relationships between compound–proteome interaction signatures to predict genome- and indication-specific drug regimens for particular individuals in a shotgun and holistic manner (i.e., against all indications simultaneously). To assess and improve the accuracy of our platform, we collaborate with experimental investigators for preclinical and clinical validation of our top ranking drug candidates (see Figure 1). The experimental results obtained are integrated back into the modeling platform to iteratively improve its accuracy.

Our polyphmarcological approach also has utility in overcoming the development of the drug-resistant strains of pathogens. Evidence has been accumulating to suggest a role for underlying multiple pathways acting in a disease-specific manner in response to a synthetic agent, to cause specific mutations for drug resistance [23]. This synthetic lethality and resistance may be overcome by designing drugs to work in a disease-specific polypharmacological manner by taking into account an interactome of multiple disease pathways and drug interactions. Using computational screening to assess multitarget binding and inhibition, our platform relies on an interaction signature of how well a compound interacts with a library of protein structures that are considered representative of the (current) structural universe, compared with how that individual compound interacts with a specific protein. As a result, multiple drugs with a therapeutic effect towards a disease process can be identified. Multiple drugs used together (polypharmacy) will reduce the occurrence of drug resistance since the simultaneous occurrence of multiple mutations that are resistant to a drug combination are exponentially less prevalent [24,25]. Polypharmacy may strengthen the effect, leading to the requirement for decreased therapeutic doses of individual compounds, so that less efficacious and slightly more toxic compounds can be used safely and synergistically to achieve the desired efficacy profile.

In this work, we integrate computational docking predictions done on a proteomic scale with results from in vitro screening studies against EVD to select and prioritize compounds for further in vivo and clinical testing.

## 2. Results

Table 1 lists selected top consensus multitarget drug candidate predictions against Ebola generated by the CANDO platform that have not yet been validated at the bench or in clinical trials. These compounds may represent potential therapeutic agents to be further investigated for in vitro and in vivo efficacy using Collaborative Cross mice [26]. Prospective in vitro validation for the top 10–100 ranked compounds at the bench (or combinations thereof) is necessary to achieve the highest likelihood of success. A comparative analysis showed that our top ranked drug candidates overlapped with 22 out of 53 (~42%) drugs identified by Kouznetsova et al. [27] (Table 2) and 24 out of 80 (30%) “FDA approved actives” identified by Johansen et al. [28] (Table 3) towards treatment of EVD. A total of 9 candidates were common between all three studies (Table 2 and Table 3).

In addition to the drugs listed in Table 2 and Table 3, predictions made by the CANDO platform had 8 overlaps (~8%) when compared to a list of 95 active compounds identified by Kouznetsova et al. with an IC_50_ of 10 to 30 µM, selectivity index less than 10 fold, or not US FDA approved for human use. These include astemizole, carvedilol, clocapramine, desloratadine, ebastine, fluspirilene, mesoridazine, and pamicogrel. Similarly, our predictions had 6 overlaps (7%) when compared to a list of 90 active compounds listed by Johansen et al. as not US FDA approved. These include alverine, astemizole, diethylstilbestrol, homochlorcyclizine, lomerizine, and tibolone. Finally, our predictions had 7 overlaps (astemizole, atovaquone, azacitidine, clemastine, clomifene, lomerizine, and sertraline) when compared to a list of 30 compounds (US FDA approved and not approved) that were prioritized by Johansen et al. as having antiviral activity in both Vero E6 and human HepG2 cell lines. One of the drug candidates common to all three studies (sertraline) resulted in statistically significant survival benefits from treatments of infected mice done by Johansen et al. [28].

**Figure 2:**
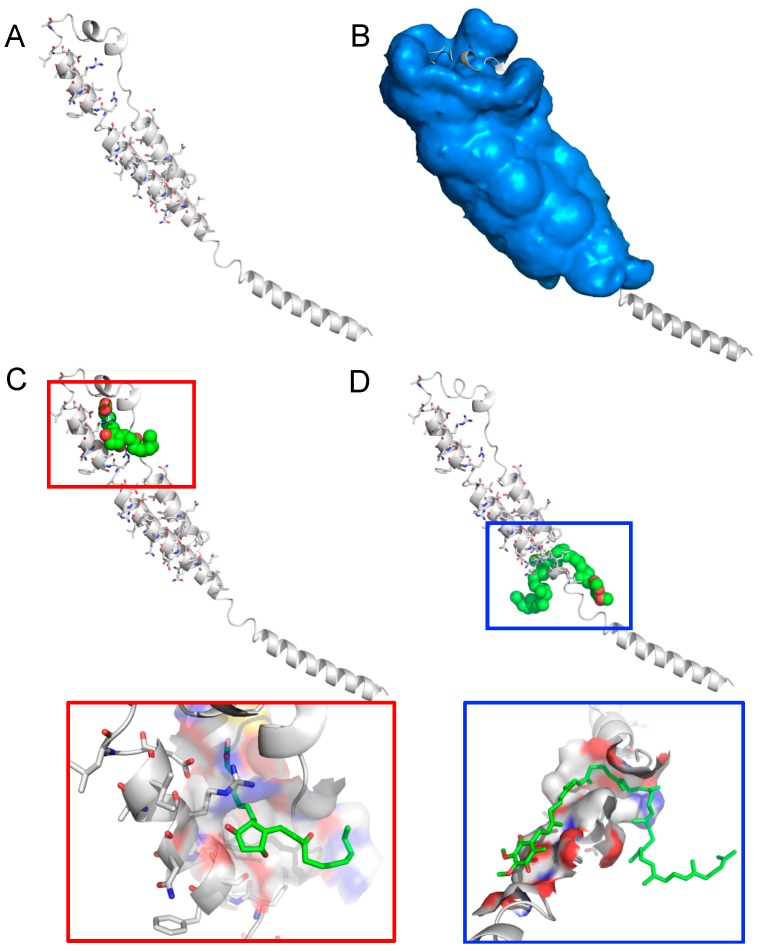
Binding modes of unoprostone and ubidecarenone to the membrane-fusion subunit from the Ebola virus envelope glycoprotein, GP2 (PDB identifier 1ebo, chain F). (**A**) Binding site residues (stick representation) predicted by COFACTOR by comparing binding motifs to a library of the PDB ligand-bound structures; (**B**) Search space for CANDOCK to dock fragments of small molecule compounds that are reconstructed while incorporating flexibility of both small molecule and the protein; (**C**) Docked conformation of uniprostone bound to the region of the fusion peptides forming disulfide-bonded loop that is homologous to an immunosuppressive sequence in retroviral glycoproteins. Uniprostone is shown as spheres (top) along with its interaction to the protein surface up to 10 Å (bottom); (**D**) Docked conformation of ubidecarenone bound to coiled coil region near the C-terminal end that acts as the membrane anchor. Ubidecarenone is shown as spheres (top) along with its interaction to the protein surface of the coiled coil region up to 10 Å (bottom). Together, these two molecules likely disrupt the conserved disulfide-bonded loop and the linker region that function as a hinge, transferring information from the GP1 receptor binding to trigger a conformational change in GP2, thereby disrupting the membrane fusion event.

Figure 2 illustrates the binding mode of two of the top drug candidates (ubidecarenone and unoprostone) to one of the common target structures (membrane-fusion subunit from envelope glycoprotein GP2, PDB identifier 1ebo, chain F) generated using our hierarchical fragment-based docking with dynamics software, CANDOCK. While this illustration enables us to rationalize the behavior of these two compounds against this particular target, we emphasize that the polypharmacological drug candidates [23] predicted by the CANDO platform are holistic in nature and do not rely on any single interaction to determine its overall efficacy (i.e., the interactions in aggregate determine efficacy). The comparative analyses between our top drug candidates against EVD and those obtained from the studies by Kouznetsova et al. and Johansen et al., as shown in Table 2 and Table 3 along with additional information on the approved indications for the known drugs and their modes of action, are discussed further below.

## 3. Discussion

Kouznetsova et al. [27] used an in vitro high-throughput screening assay to identify 53 FDA-approved drugs that block Ebola virus-like particles (VLPs) entry into cells. Johansen et al. [28] used enhanced green fluorescent protein (eGFP) activity in genetically engineered Ebola virus and cell viability studies to identify 80 FDA-approved compounds with anti-Ebola virus activity from a library of 2600 biologically active molecules. In addition, Johansen et al. prioritized 30 compounds for confirmation of antiviral activity from two *in vitro* assays from a total of 170 (US-FDA approved and non-approved) active compounds.

While both efforts move towards identifying possible treatments for EVD by screening existing drugs, there are several issues (including non-conformity in library of compounds evaluated, the selection of targets, the cell assays used, and a lack of mechanistic detail) that may limit future success for similar outbreaks. For example, Kouznetsova et al. [27] excluded certain drug categories prior to in vitro screening, including immunosuppressants, veterinary use compounds, and approved topical agents. CANDO predicted compounds from these categories to possess potential anti-EVD activity based on multitargeted inhibition of proteins from the five Ebola proteomes; however, unless preclinical vetting indicated a strong preference for their use, they would not be considered as top drug candidates against Ebola. Furthermore, Kouznetsova et al. [27] determined potential drugs using VLPs, resulting in entry or membrane fusion inhibitors that directly or indirectly block entry/fusion, interfere with glycoprotein (GP) and matrix proteins (VP24 or VP40), interact with host molecules that are involved in the fusion process, or any combination of these. The use of VLP-based assays to determine putative drugs may result in false positives, as GP, VP24, and VP40 are known to exhibit cell-specific behavior. The use of the HeLa cell line is also potentially problematic due to inherent variability and known chromothripsis [29] that may have a profound effect on viral replication. We propose the use of Huh7 cell lines as a more robust choice for screening EVD since hepatic cells are known targets for Ebola infection in vivo [30,31,32].

Analyzing the overlap between candidate drugs against EVD based on the Johansen et al. study and those generated by CANDO (Table 3), we observe that: Five drugs possess affinity to the H1 histamine receptor (antagonism) and are used for relatively similar clinical indications. Six drugs are hormonal (four estrogen, one androgen, one glucocorticoid) receptor modulators that are used to treat a variety of clinical conditions such as hormone replacement therapy, reactive airway disease. One drug is a serotonin receptor inhibitor used to treat depression and anxiety and another one is a dopamine receptor agonist used to treat pituitary tumors and Parkinson’s disease. Two drugs work by disrupting sodium-potassium transmembrane transport in the myocardium and are used to treat cardiac arrhythmias. Finally, two drugs work by disrupting the tubulin structure during cell division and are used to treat parasites and virus (HPV warts)-related conditions.

Analyzing the overlap between candidate drug against EVD based on the Kouznetsova et al. study and those generated by CANDO (Table 2), we observe the following: Five drugs act upon the cardiac myocyte cell membrane (two sodium channel blockers, two sodium-potassium channel blockers, and one potassium channel blocker) and are used to treat various cardiac arrhythmias. Two drugs affect the activity of DNA topoisomerase (I and II) and are used as chemotherapeutic agents. Three drugs work as selective estrogen receptor modulators and one drug is a selective serotonin receptor inhibitor and are used correspondingly. Finally, two drugs work by disrupting the tubulin structure during cell division and are used to treat parasites, inflammation, and related clinical scenarios.

Analyzing the nine drug candidates common to all three (CANDO, Kouznetsova et al., and Johannsen et al.; indicated by italics in Table 2 and Table 3) studies, we observe that there was one H1 histamine receptor blocker (out of five), three estrogen receptor modulator (three of six), one tubulin destabilizer, one serotonin receptor inhibitor, and two cardiac membrane channel blockers (out of five such candidates with the same mode of action across the three studies). More generally, these analyses indicate that compounds or drugs with these mechanisms of action are likely to be efficacious against EVD than others.

The putative drug candidates against EVD were selected using a consensus approach that has not yet been rigorously benchmarked and analyzed, in contrast to the CANDO signature comparison and ranking approach [18,22,23]. In addition, there are other issues not considered by CANDO that may affect its accuracy, including but not limited to considering only protein structure (as opposed to DNA and RNA) targets, not handling post-translational modifications explicitly, and not integrating dynamic information such as expression (copy number) data to evaluate the most biologically relevant proteomes.

Our preliminary results here in terms of the overlap between our study and those of Kouznetsova et al. [27] and Johansen et al. [28] indicates promise in using our overall approach to selecting efficacious drugs that are effective in vivo (for example, sertraline resulted in statistically significant survival benefits from treatments of infected mice done by Johansen et al.). However, the preclinical and clinical protocols to evaluate these drug candidates will need to be refined keeping these limitations in mind, especially since CANDO is designed to work by compound–proteome signature comparisons, and finding hits against small viral proteomes may necessitate a modified approach tailored to specific indications.

## 4. Materials and Methods

We have developed the Computational Analysis of Novel Drug Opportunities (CANDO) platform based on the hypothesis that drugs function by interacting with multiple protein targets to create a molecular interaction signature that can be exploited for therapeutic repurposing and discovery. The large number of methods, protocols, and pipelines that comprise this integrated platform are described in detail elsewhere [18,22,23].

We compiled a library of 3733 compounds that are human ingestible with established side effects (FDA-approved drugs), followed by a hierarchical fragment-based multitarget docking with dynamics screen against a large (48,278) library of experimentally determined and modeled protein structures to construct compound–proteome interaction matrices that were then analyzed to determine similarity in drug behavior. Initially, a structure modeling and docking pipeline is used to model the structures of all proteins whose structures are not available in the Protein Data Bank (PDB) [33]. The rough poses of compound–proteome interactions are determined using chem- and bioinformatics methods and hierarchically refined using fragment-based docking with dynamics simulations of all the atoms in the system (currently implemented by the CANDOCK software), which we have shown previously to be necessary for the accurate calculation of binding energies [34,35]. The integrated modeling pipeline uses HHBLITS [36], ITASSER [37,38], and KoBaMIN [39,40,41] for protein modeling, refinement, and dynamics, and COFACTOR [42] for the identification of ligand binding sites. The protocols used for modeling the Ebola proteomes and the generation of the Ebola compound–proteome interaction matrices, along with the details of the protein structure modeling, binding site identification, parameter selection and optimization, compound–proteome interaction-signature generation and comparison, compound ranking, and accuracy calculation are exactly as described in the methods section of [22]. The CANDO platform is agnostic to the docking or interaction determination method used, and recent comparative studies conducted by us indicate that using other publicly available methods for docking also produce similar outcomes with varying benchmarking accuracies (unpublished). This is further supported by our successful initial shotgun multitarget drug repurposing studies that used a different docking with dynamics protocol from what is currently used by the CANDO platform, namely Autodock for docking and NAMD for dynamics simulation [15,16].

The proteomic signature similarity of drugs is then used to rank putative drug candidates for all indications in a shotgun manner. We have used the CANDO platform to generate putative drug candidate rankings for all 2030 indications with at least one approved drug and to perform rigorous benchmarking for 1439 indications with two or more approved drugs. Benchmarking performance varies from 12%–40% depending on the number of top ranking drug candidates and indications considered (in contrast to high-throughput and random screening rates of 0.2%). We conducted thirteen prospective validation studies covering ten diseases (including dengue, dental caries, diabetes, hepatitis B, herpes, lupus, malaria, and tuberculosis), with 58/163 (~35%) drugs showing better activity than the standard clinical-use drug [18].

Figure 1 illustrates the application of the CANDO repurposing platform to generate putative drug candidates predicted to inhibit multiple protein targets encoded by the genomes of five Ebola virus strains: *Zaire ebolavirus*, *Sudan ebolavirus*, *Taï Forest ebolavirus*, *Bundibugyo ebolavirus,* and *Reston ebolavirus* (*R. ebolavirus* is not infective to humans) [4]. The structures of all the proteins from different strains available in the PDB were used, (e.g., 1ebo-F). All other structures in different viral strains were modeled and the interactions generated using the pipeline described above. The drug–protein interactions were ranked according to their corresponding scores (ranging from 0–2) and filtered using a specific cutoff to eliminate those with a low score. The interaction scores were calculated exactly as described in the methods section of [22] and are a pseudo measure corresponding to binding strength, with higher scores indicating stronger interactions. Cutoffs for this score are determined based on parameterization of the platform to known drug–compound interactions from the PDB.

The remaining top ranked candidates were further clustered to determine the consensus score, which represents the frequency of occurrence of each compound interacting with different protein targets (since a compound may interact with multiple proteins from different strains) as shown in Table 1. We integrated the top ranked putative drug candidates with in vitro hits previously identified in bench studies [28,27] to identify common leads that may be pursued in vivo and in clinical trials. Putative drug candidates with high interaction score (>= 1.1 cutoff ) and high consensus score (Table 1) and in vitro screening overlaps (Table 2 and Table 3) are suggested as putative treatments for future EVD outbreaks.

## 5. Conclusions

Aside from top ranking drug candidates generated by the CANDO platform which have not yet been experimentally validated in vitro, there are 22 compounds that overlap between CANDO and drugs identified by Kouznetsova et al. [27], with 10 drugs with high scoring interactions (Table 2). Similarly, there are 24 compounds that overlap between CANDO and FDA approved drugs listed by Johansen et al. [28] study, with 11 drugs with high scoring interactions (Table 3). This demonstrates that computational methods can accurately and efficiently identify potential leads for further evaluation individually or as drug combinations, inhibiting different protein targets to devise potent therapies against EVD. Future work would include experimentally evaluating the suggested combination therapy in vitro and, if successful, moving to Phase I clinical trials.

## Figures and Tables

**Table 1:** Selected top ranked drug candidates against Ebola generated by the CANDO platform.

Compound(s)	Interaction Score	Consensus Score (min)	Protein Target Identifiers
enfuvirtide	2.0	7	GP2, VP35, 1ebo-F
vancomycin, bleomycin	2.0	10	GP1,2, pre-sGP, SGP, SsGP
octreotide, lanreotide, somatostatin	2.0	10	GP1,2, pre-sGP, SGP, SsGP
ubidecarenone (CoQ10)	1.6	7	GP1,2, GP2, VP24, VP35, VP40, 1ebo-F
unoprostone	1.3	10	GP1,2, VP35, VP24, 1ebo-F

The name of the compound, a measure of its binding strength or interaction score (range 0-2), its frequency of occurrence or consensus score, and the Uniprot short names or PDB identifiers of the protein targets that it binds to are given. The protein targets are GP2—envelope glycoprotein; VP35—polymerase cofactor VP35; 1ebo-F—membrane-fusion subunit from envelope glycoprotein GP2; pre-sGP—pre-small secreted glycoprotein; sGP—secreted glycoprotein; SsGP—super small secreted glycoprotein; and VP24—membrane-associated protein VP24. A combination of drugs that have broad specificity and/or are derived from disparate functional classes (for example: enfuvirtide and ubidecarenone (CoQ10) AND vancomycin OR octreotide/lanreotide/somatostatin) may be the most promising combinations to pursue for further preclinical and clinical validation.

**Table 2:** Overlap between drug candidates identified by the CANDO platform and those identified by Kouznetsova et al. [27].

Compound(s)	Interaction Score	Consensus Score	Approved Indication	Mode of Action
Niclosamide *	1.897	3	helminthic infestation	inhibits parasite metabolism
Sertraline *	1.897	1	depression, anxiety	selective serotonin receptor inhibitor
Clomifene *	1.897	1	anovulation, oligoovulation	selective estrogen receptor modulator
Alverine	1.897	1	gastrointestinal muscle spasms	parasympathetic nervous system modulator
Aprindine	1.897	1	cardiac arrhythmia	sodium channel inhibitor
Mebendazole *	1.897	1	helminthic infestation	tubulin destabilizer
Salmeterol	1.890	2	asthma	beta 2 adrenergic receptor agonist
Topotecan	1.823	1	ovarian and lung cancers	DNA topoisomerase I inhibitor
Deslanoside *	1.377	10	cardiac arrhythmia	sodium-potassium channel blocker
Propafenone	1.298	10	cardiac arrhythmia	sodium channel blocker
Digoxin *	1.067	1	cardiac arrhythmia	sodium-potassium channel blocker
Proglumetacin	1.058	1	non-steroidal anti-inflammatory drug	cyclooxygenase-1 inhibitor
Posaconazole	1.023	3	fungal infection (aspergillus and candida)	membrane bound enzyme inhibitor
Raloxifene *	0.843	2	osteoporosis and breast cancer prevention	selective estrogen receptor modulator
Clarithromycin	0.741	2	bacterial infections	protein synthesis inhibitor
Clemastine *	0.741	1	allergies	H1 histamine receptor inhibitor
Colchicine	0.741	1	gout, pericarditis	microtubule inhibitor
Tamoxifen *	0.741	1	estrogen receptor positive breast cancer	Selective estrogen receptor modulator
Thiothixene	0.741	1	psychotic disorders, e.g., schizophrenia	dopamine antagonist
Daunorubicin	0.714	1	hematologic dyscrasia (acute lymphocytic leukemia, acute myeloid leukemia)	DNA topoisomerase II inhibitor
Dronedarone	0.722	1	cardiac arrhythmia	potassium channel blocker
Vincristine	0.707	1	hematologic dyscrasia (acute lymphocytic leukemia, acute myeloid leukemia)	microtubule inhibitor

* Drugs also identified by Johansen et al. [28].

**Table 3:** Overlap between drug candidates generated by the CANDO platform and those identified by Johansen et al. [28] as FDA approved actives.

Compound(s)	Interaction Score	Consensus Score	Approved Indication	Mode of Action
Niclosamide *	1.897	3	helminthic infestation	inhibits parasite metabolism
Quinestrol	1.897	3	hormone replacement therapy	synthetic steroidal estrogen receptor agonist
Sertraline *	1.897	1	depression, anxiety	selective serotonin receptor inhibitor
Clomifene *	1.897	1	anovulation, oligoovulation	selective estrogen receptor modulator
Propoxyphene	1.897	1	mild to moderate pain	opiate receptor binder
Atovaquone	1.897	1	pneumocystis Pneumonia	dihydroorotate dehydrogenase inhibitor
Azelastine	1.897	1	allergic rhinitis	H1 histamine receptor inhibitor
Danazol	1.897	1	endometriosis	androgen receptor competitive inhibitor
Mebendazole *	1.897	1	helminthic infestation	tubulin destabilizer
Hydroxyprogesterone	1.823	2	preterm labor	steroidal progesterone receptor agonist
Deslanoside *	1.377	10	cardiac arrhythmia	sodium-potassium channel blocker
Digoxin *	1.067	1	cardiac arrhythmia	sodium-potassium channel blocker
Ritonavir	1.057	2	HIV infection	protease inhibitor
Raloxifene *	0.843	2	osteoporosis and breast cancer prevention	selective estrogen receptor modulator
Ciclesonide	0.843	1	asthma, allergic rhinitis	glucocorticoid receptor agonist
Clemastine *	0.741	1	allergies	H1 histamine receptor inhibitor
Podofilox	0.741	1	skin warts caused by Human papilloma virus	tubulin polymerization inhibitor
Tamoxifen *	0.741	1	estrogen receptor + breast cancer	selective estrogen receptor modulator
Desloratadine	0.741	1	allergies	H1 histamine receptor inhibitor
Methdilazine	0.741	1	allergy symptoms, antiemetic	H1 histamine receptor antagonist
Chlorcyclizine	0.741	1	allergy symptoms, antiemetic	H1 histamine receptor antagonist
Azacitidine	0.712	1	myelodysplastic syndrome	DNA methyl transferase inhibitor
Terconazole	0.711	1	fungal infection	ERG11/CYP51 inhibitor
Bromocriptine	0.707	1	pituitary tumors, Parkinson’s disease	dopamine receptor agonist

* Drugs also identified by Kouznetsova et al. [27].
